# The role of vascular invasion and lymphatic invasion in predicting recurrent thoracic oesophageal squamous cell carcinoma

**DOI:** 10.1186/s12957-021-02458-1

**Published:** 2022-01-10

**Authors:** Yu Ma, Xi Yao, Zhenzhen Li, Jie Chen, Wensheng Li, Hongtao Wang, Lanjun Zhang, Jianfei Zhu

**Affiliations:** 1 Department of Pathology, Shaanxi Provincial People’s Hospital, No. 256 Youyi Road West, Xi’an, 710068 Shaanxi People’s Republic of China; 2 Department of Anesthesiology, Shaanxi Provincial People’s Hospital, No. 256 Youyi Road West, Xi’an, 710068 Shaanxi People’s Republic of China; 3 Department of Thoracic Surgery, Shaanxi Provincial People’s Hospital, No. 256 Youyi Road West, Xi’an, 710068 Shaanxi People’s Republic of China; 4 Department of Thoracic Surgery, Sun Yat-sen University Cancer Center, No. 561 Dongfeng Road East, Guangzhou, 510060 Guangdong People’s Republic of China

**Keywords:** Oesophageal squamous cell carcinoma, Lymphatic invasion, Vascular invasion, Disease-free survival, Lymph node metastasis

## Abstract

**Background:**

Numerous studies have addressed lymphovascular invasion (LVI) in patients with thoracic oesophageal squamous cell carcinoma (ESCC); however, little is known about the individual roles of lymphatic invasion (LI) and vascular invasion (VI). We aimed to analyse the prognostic significance of LI and VI in patients with thoracic ESCC from a single centre.

**Methods:**

This retrospective study included 396 patients with thoracic ESCC who underwent oesophagectomy and lymphadenectomy in our hospital. The relationship between LI, VI and the other clinical features was analysed, and disease-free survival (DFS) was calculated. Survival analysis was performed by univariate and multivariate statistics.

**Results:**

Briefly, VI and LI were present in 25.8% (102 of 396) and 23.7% (94 of 396) of ESCC patients, respectively, with 9.15% patients presenting both LI and VI; the remaining patients did not present LI or VI. We found that LI was significantly associated with pN stage (*P*<0.001) and pTNM stage (*P*<0.001), and similar results were found in VI. Moreover, survival analysis showed that pT stage (*P*<0.001), pN stage (*P*=0.001), pTNM stage (*p*<0.001), VI (*P*=0.001) and LI (*P*<0.001) were associated with DFS in ESCC. Furthermore, multivariate analysis suggested that pT stage (RR=1.4, *P* =0.032), pN stage (RR=1.9, *P*<0.001) and LI (RR=1.5, *P*=0.008) were independent predictive factors for DFS. Finally, relapse was observed in 110 patients (lymph node metastasis, 78 and distant, 32) and 147 patients with cancer-related deaths. Subanalysis showed that LI-positive patients had higher lymph node metastasis, although there was no significant difference (32.1% vs. 15.6%, *P*=0.100).

**Conclusions:**

LI and VI were common in ESCC; they were all survival predictors for patients with ESCC, and LI was independent. Patients with positive LI were more likely to suffer lymph node metastasis.

## Introduction

Worldwide, oesophageal cancer is the seventh most widespread cancer and constitutes the sixth leading cause of cancer death, accounting for 5.3% of all global cancer deaths [1]. Although some studies have indicated an incidence reduction in the last few decades, oesophageal cancer is a significant public health burden in China [2]. Recently, the rate of superficial oesophageal squamous cell carcinoma has been increasing because of advances in endoscopic diagnosis and treatment; even so, the prognosis remains poor [3, 4]. Several studies have evaluated risk factors for cancer recurrence and survival. Age, tumour length, tumour width, TNM staging, surgical approach, perineural invasion and lymphovascular invasion were significantly associated with locoregional recurrence and distant metastasis after oesophagectomy [5–7]. Moreover, lymphovascular invasion (LVI) has been reported to be a critical pathologic feature of metastasis in urothelial carcinoma, colorectal cancer and several kinds of solid carcinoma [8–11]. Although the staging guidelines of the American Joint Cancer Committee on Cancer mandated distinguishing lymphatic and vascular invasion (LI vs. VI) as early as 2005, these guidelines lack a routine standard and objective assessment method to reliably differentiate them [12]. Some studies have reported that immunohistochemistry (IHC) staining appears more reliable to distinguish lymphatic and vascular invasion than haematoxylin and eosin (H&E) staining [13–15]. Previous studies focused on LI and VI together in patients with thoracic oesophageal squamous cell carcinoma (ESCC); however, the data on the individual role of LI and VI were limited. The aim of the present study was to analyse the individual influence of LI and VI on the prognosis of ESCC.

## Methods

### Patients selection

**Fig. 1:**
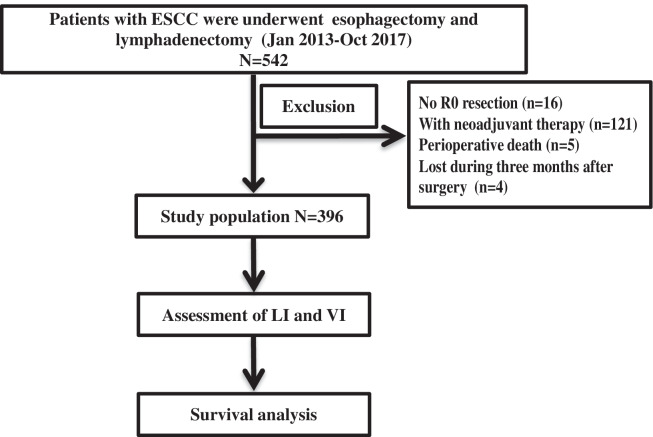
The flow diagram of this study

A retrospective study was performed on 396 patients who underwent oesophagectomy and lymphadenectomy in Shaanxi Provincial People’s Hospital between Jan. 2013 and Oct. 2017 (Fig. 1). The patients included in the current study met the following criteria: (1) diagnosis of squamous cell carcinoma; (2) complete resection and regular follow-up; (3) no neoadjuvant therapy; (4) no distant metastasis; (5) no other malignant cancer; and (6) immunohistochemical staining of tissues during diagnosis. The patients were followed up until October 2020 by systematic physical examination and standard laboratory screening every 6 months to evaluate tumour recurrence and metastasis.

### Pathological evaluation

All surgical specimens were processed according to standard pathological procedures. Briefly, the gross specimens and lymph nodes were fixed in 10% neutral buffered formalin immediately after collection and subsequently embedded in paraffin. Serial sections at 4 μm thickness were stained with H&E. Pathological staging was based on the eighth edition of the American Joint Committee on Cancer TNM staging system [16]. All pathologists were blinded to the clinical outcomes.

### Immunohistochemical staining

Two consecutive oesophageal carcinoma sections from each patient were stained with monoclonal antibodies against CD31 and D2-40 (ready-to-use, Maixin, China) to assess blood vessels and lymphatic vessels, respectively. Immunohistochemical staining was performed by using an automated immunostainer and an Ultra View Universal DAB (3,3′-diaminobenzidine) Detection Kit (Ventana Medical Systems, Inc. Tucson, AZ, USA). Two full sections of tonsil tissue were used as positive and negative controls for each antibody. All microscopic analyses were carried out using a light microscope (Zeiss, Germany).

### Assessment of LI and VI

**Fig. 2:**
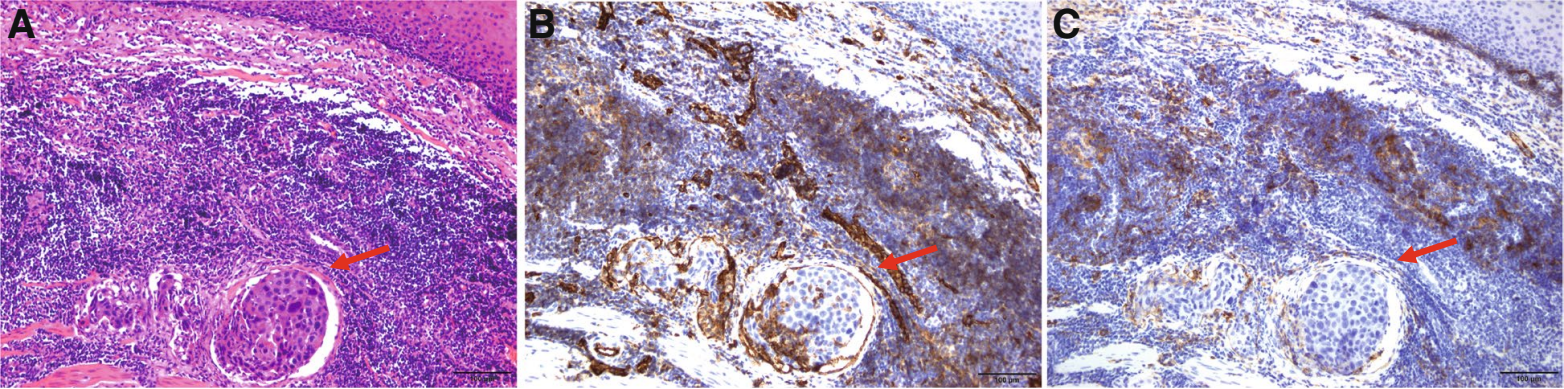
Representative histology of vascular invasion in oesophageal squamous cell carcinoma. **A** H&E staining of conspicuous carcinoma emboli in vascular space (red arrow). **B** Vascular vessel stained positive for CD31. **C** Vascular vessel stained negative for D2-40

**Fig. 3:**
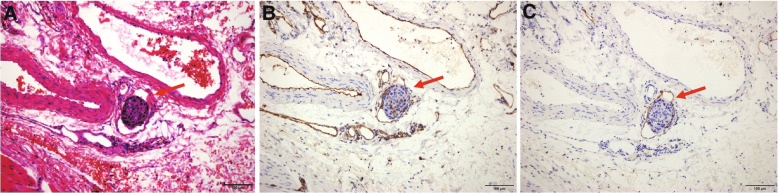
Representative histology of lymphatic invasion in oesophageal squamous cell carcinoma. **A** H&E staining of conspicuous carcinoma emboli in lymphatic vessel space (red arrows). **B** Lymphatic vessel stained positive for CD31. **C** Lymphatic vessel stained positive for D2-40

Previous studies have confirmed that neoadjuvant therapy can affect the evaluation of LVI after oesophagectomy [4, 17], so we excluded all the specimens of these patients receiving neoadjuvant therapy. H&E staining of LVI sections was reviewed blinded to the pathological report and evaluated by the attending pathologists. For the assessment of LI and VI, separate sections from the same paraffin block were stained with CD31 and D2-40. The peritumoural and invasive fronts and intratumoural areas were counted. LVI on H&E-stained sections was identified as the presence of tumour cell emboli within the endothelium-lined spaces. VI was counted only when tumour cells were positive for CD31 and negative for D2-40 (Fig. 2). As CD31 can also stain part of the lymphatic vessels, LI was defined as the lumen where the tumour embolus was located and positively stained with CD31 and D2-40 at the same time (Fig. 3). All specimens were randomly chosen and observed by the different pathologists who were blinded to the previous results. Any inconsistencies were re-evaluated by all the pathologists until a consensus was reached.

### Statistical analysis

The correlation between LVI and clinicopathological features was tested by chi-square test. The Kaplan–Meier method was used to calculate disease-free survival (DFS), and differences were assessed with the log-rank statistic. All statistically significant prognostic factors identified in the univariate analysis were included in the Cox regression multivariate analysis. *P* value < 0.05 was considered to indicate statistical significance. Analyses were performed with SPSS (version 22.0).

## Results

### Clinical and pathological characteristics of included patients

The median age of the patients was 65 years (range: 42 to 87), and 73.2% of patients were men (290/396). In total, VI was present in 25.8% of patients (102 of 396), and LI was present in 23.7% of patients (94 of 396). Among the above patients, 36 patients presented both LI and VI (9.1%), while the remaining patients did not present LI or VI. The relationships between lymphatic/vascular invasion and clinicopathological characteristics are listed in Table 1. VI was significantly associated with pT stage (*P*=0.013), pN stage (*P*<0.001) and pTNM stage (*P*<0.001), but not with gender, age, smoking status, tumour location, tumour length, surgical approach or differentiation. The only difference between LI and VI was that LI was not associated with pT stage (*P*=0.376). In detail, LI was present in 20.8% (26/125) of patients in T1+T2 and 25.1% (68/271) of patients in T3+T4.

**Table 1:** The relationship between lymphovascular invasion and clinicopathological features of oesophageal squamous cell carcinoma

Variable	Total	Vascular invasion	*P* value	Lymphatic invasion	*P* value
	396	Presence(%)		Presence(%)	
Gender			0.437		0.427
Male	290	78 (26.9)		72 (24.8)	
Female	106	24 (22.6)		22 (20.8)	
Age			0.766		0.759
<65 years	325	85 (26.2)		76 (23.3)	
≥65 years	71	17 (23.9)		18 (25.4)	
Smoking status			0.630		0.537
Non-smoker	138	33 (23.9)		30 (21.7)	
Smoker	258	69 (26.7)		64 (24.8)	
Tumour location			0.347		0.671
Upper	35	7 (20.0)		8 (22.9)	
Middle	253	62 (24.5)		57 (22.5)	
Lower	108	33 (30.6)		29 (26.9)	
Tumour length			1.000		0.637
≤4.0 cm	199	51 (25.6)		45 (22.6)	
>4.0 cm	197	51 (25.9)		49 (24.9)	
Surgical approach			1.000		0.897
Right incision	118	30 (25.4)		27 (22.9)	
Left incision	278	72 (25.9)		67 (24.1)	
Differentiation			0.274		0.997
G1	119	37 (31.1)		28 (23.5)	
G2	184	44 (23.9)		44 (23.9)	
G3	93	21 (22.6)		22 (23.7)	
p T stage			0.013		0.376
T1+T2	125	22 (17.6)		26 (20.8)	
T3+T4	271	80 (29.5)		68 (25.1)	
p N stage			<0.001		<0.001
N0	210	32 (15.2)		33 (15.7)	
N1+N2+N3	186	70 (37.6)		61 (32.8)	
p TNM stage			<0.001		<0.001
I stage	49	4 (8.2)		6 (12.2)	
II stage	164	29 (54.9)		25 (15.2)	
III stage	170	60 (35.3)		55 (32.4)	
IV stage	13	9 (69.2)		8 (61.5)	
Relapse type			0.816		0.100
Lymph node recurrence	78	23 (29.5)		25 (32.1)	
Metastasis	32	8 (25.0)		5 (15.6)	

### Survival analysis

**Fig. 4:**
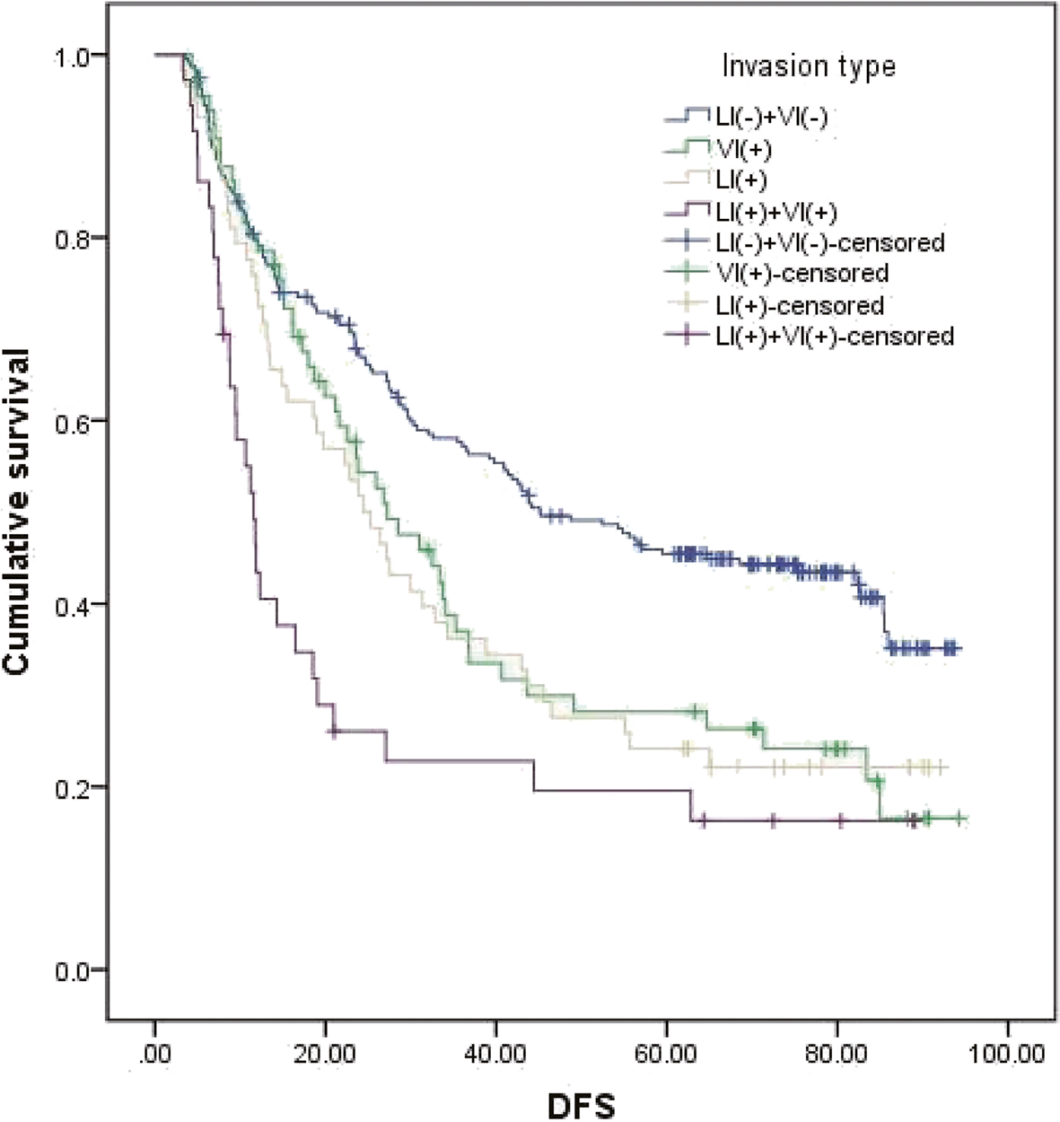
Disease-free survival according to the type of lymphovascular invasion

**Fig. 5:**
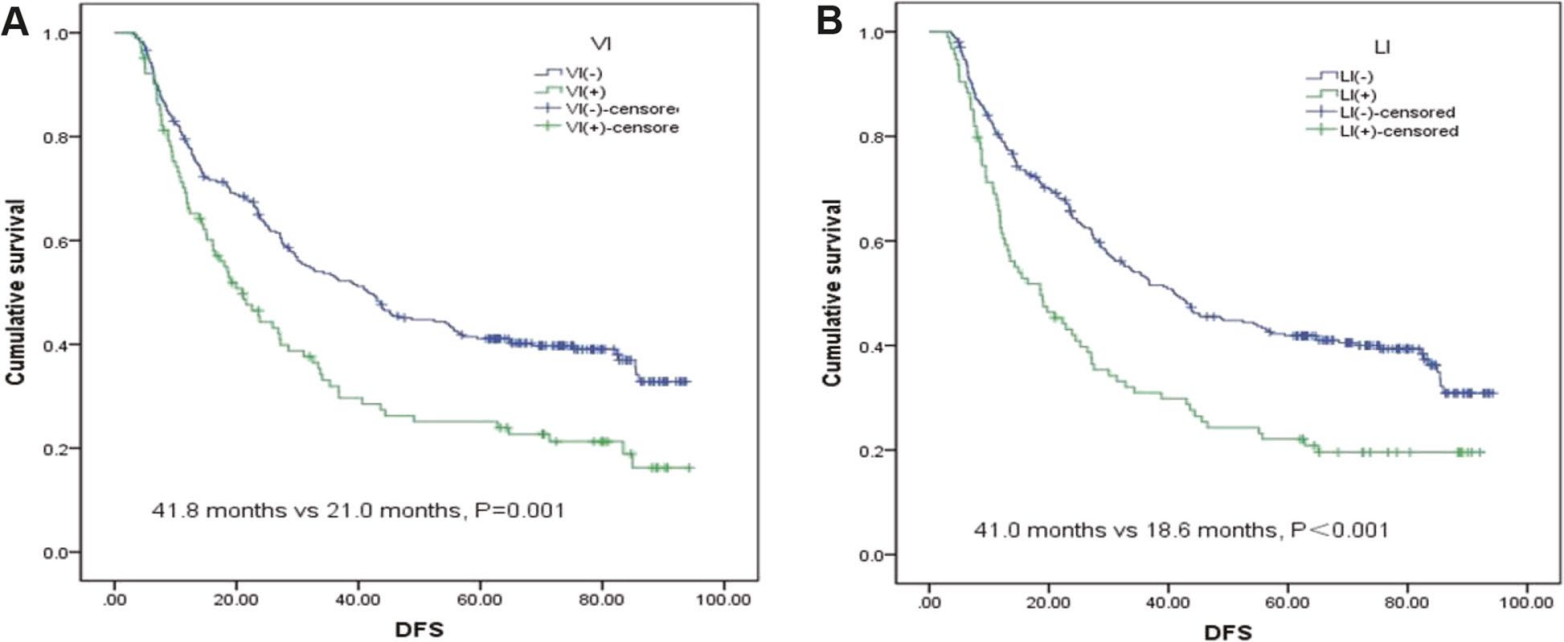
Disease-free survival according to the individual roles of vascular invasion and lymphatic invasion. **A** Disease-free survival according to the individual role of vascular invasion. **B** DFS according to the individual role of lymphatic invasion

In Table 2, the predictors for DFS of patients with ESCC by univariate analysis are listed. Briefly, pT stage (*P*<0.001), pN stage (*P*=0.01), pTNM stage (*P*<0.001), VI (*P*=0.01) and LI (*P*<0.001) were associated with DFS in ESCC. Among all patients, the prognosis of patients was the worst in those with double positivity (VI and LI) and the best for patients with double negativity (VI and LI); however, there was no significant difference in individual LI positivity and VI positivity (45.1 months vs. 27.2 months vs. 24.5 months vs. 11.6 months, respectively; *P*<0.001) (Fig. 4). When reviewing DFS, both LI and VI were predictors of survival (LI: DFS 41.0 months vs. 18.6 months, *P*<0.01; VI: DFS 41.8 months vs. 21.0 months, *P*=0.001) (Fig. 5).

**Table 2:** Predictors for disease-free survival in oesophageal squamous cell carcinoma by univariate analysis

Variable	Median	Disease-free survival (months)	*P* value
		95% CI	
Gender			0.029
Male	29.2	23.1–35.3	
Female	42.9	22.4–63.3	
Age			0.608
<65 years	34.2	26.6–41.9	
≥65 years	27.2	20.5–33.9	
Smoking status			0.064
Non-smoker	43.7	20.9–66.4	
Smoker	28.7	22.6–34.8	
Tumour location			0.379
Upper	27.1	20.7–33.5	
Middle	39.2	31.1–47.2	
Lower	27.2	20.9–33.5	
Tumour length			0.003
≤4.0 cm	42.9	34.4–51.3	
>4.0 cm	27.4	23.0–31.8	
Surgical approach			0.734
Right incision	27.2	19.3–35.0	
Left incision	35.3	27.7–42.8	
Differentiation			0.763
G1	30.0	19.7–40.3	
G2	32.8	22.0–43.6	
G3	35.5	21.0–49.8	
p T stage			<0.001
T1+T2	56.3	28.3–84.3	
T3+T4	27.1	22.6–31.5	
p N stage			<0.001
N0	68.5	45.4–91.6	
N1+N2+N3	19.7	13.9–25.5	
Adjuvant therapy			0.570
With	38.8	29.4–48.2	
Without	27.8	22.4–33.1	
p TNM stage			<0.001
I stage	71.4	43.9–98.8	
II stage	56.7	31.1–82.3	
III stage	21.6	15.7–27.5	
IV stage	10.8	4.9–16.6	
Vascular invasion			0.001
Presence	21.0	15.5–26.5	
No	41.8	32.1–51.6	
Lymphatic invasion			<0.001
Presence	18.6	11.1–26.1	
No	41.0	31.8–50.2

### Independent factors affect DFS of ESCC

A multivariate analysis using the Cox proportional hazard model for all the patients showed that pT stage (RR=1.4, *P*=0.032), pN stage (RR=1.9, *P*<0.001) and LI (RR=1.5, *P* = 0.008) were predictive factors for prognosis with regard to DFS. Meanwhile, VI (RR=1.3, *P*=0.086) was not a prognostic factor. This suggests that individual LI, but not LVI or individual VI, is a dependent prognostic factor for ESCC (Table 3).

**Table 3:** Independent factors affecting disease-free survival in oesophageal squamous cell carcinoma by multivariate analysis

Variable	RR	Disease-free survival	*P* value
		95% CI	
Gender: female/male	0.8	0.6–1.1	0.260
Tumour length:≤4.0cm/>4.0cm	1.2	0.9–1.5	0.171
Differentiation:G1/G2/G3	1.0	0.8–1.1	0.595
p T stage:T1+T2/T3+T4	1.4	1.0–1.8	0.032
p N stage:N0/N1+N2+N3	1.9	1.5–2.5	<0.001
Vascular invasion: no/presence	1.3	1.0–1.7	0.086
Lymphatic invasion: no/presence	1.5	1.1–1.9	0.008

### Recurrence and metastasis modes of ESCC

The median follow-up time was 57.3 months (range: 4.9–94.2). Loss to follow-up was 7.8% (31/296). Relapse was observed in 110 patients (lymph node metastasis 78 or distant 32); 141 patients were still alive at the last follow-up, and 147 patients had cancer-related deaths. Subanalysis showed that LI-positive patients had higher lymph node metastasis, although there was no significant difference (32.1% vs. 15.6%, *P*=0.100). The data are summarized in Table 1.

## Discussion

Several previous studies have shown that LVI influences the prognosis of ESCC [18–20]. Hsu CP et al. [4] considered that the prognostic impact of LVI was primarily in the subgroup of node-negative patients who received primary oesophagectomy. Similarly, Huang et al. [21] indicated that in patients who underwent primary oesophagectomy, LVI was associated with poor disease-specific survival or disease-free survival, and they believed that LVI may precede or occur concurrently with lymph node metastasis. In the current study, the results were in accordance with their findings, and both LI and VI were predictors of survival.

Moreover, most of the previous studies combined LI and VI together and investigated the effects of prognosis on lymphatic and vascular invasion together [22–24]. Individual studies of LI and VI are scattered. A previous study suggested that angiogenesis and lymphatic vessel formation play different roles in the early stages of tumour formation, and they found that lymphangiogenesis may promote the initial metastatic process of nonseminomatous testicular germ cell tumours rather than angiogenesis and blood vascular invasion [25]. Additionally, Spoerl S et al. [26] reported significantly lower OS and RFS in patients with LI (OS: 41.1%, RFS: 38.3%) in contrast to patients with LI-negative oral squamous carcinoma (OS: 66.8%, RFS: 59.7.7%, *P* < 0.001). However, there were limited similar studies on ESCC. In the current study, LI and VI were predictors of survival for patients with ESCC, and LI was an independent factor, indicating that LI might play an important role in ESCC.

The Japan Esophageal Society guideline of 2017 recommended that endoscopic resection (ER) can be applied for lesions that infiltrate the muscularis mucosae or inner submucosa (T1b-SM1), but the risk of lymph node metastasis still exists for these cases. Deeper superficial carcinomas (T1b-SM2 and T1b-SM3) should not be treated with endoscopy alone due to the high rates of metastasis [27, 28]. Different observations have been made in the literature concerning the possible reasons. J. Oguma et al. [29] hypothesized that lymphatic invasion occurs first during invasion from the muscularis mucosae to the upper layer of the submucosa, and venous invasion may occur after lymphatic invasion as the tumour invades deeper layers of the submucosa. The mechanical stress generated by proliferating tumour cells and high interstitial fluid pressure may affect intratumour vascular function [25]. LI seems to be more affected by these processes than VI, which might be due to the thin endothelial wall and missing basal membrane of the lymph vessels [25, 30].

In a review of previous literature, the majority of methods for identifying LVI were based on H&E staining. However, some studies have used special biomarkers for the lymphatic and vascular endothelia to accurately detect and distinguish lymphatic and vascular invasion. Faiz et al. [31] identified the type of vascular invasion of tumour cells by performing additional Elastica van Gieson staining to confirm or exclude the presence of extramural venous invasion. In this study, the recognition of LI and VI was based on immunohistochemical staining with an anti-D2-40 antibody for lymph vessels and an anti-CD31 antibody for blood vessels. In some cases diagnosed as LVI negative on H&E staining, immunostaining discerned the retraction artefacts and confirmed the existence of lymphatic vessels when a tumour embolus completely obliterated the lumen of the lymphatic channel [32, 33]. In our experience, immunohistochemistry has been routinely used in the Pathology Department quickly and effectively at our institution.

Postoperative recurrence of oesophageal cancer is also a concern. Our team found that local recurrence was twice as common as distant recurrence in the first 3 years after oesophagectomy, and the type of local recurrence was mainly lymph node metastasis [34]. Yang and his colleague conducted a meta-analysis of the relationship of LVI with lymph node metastasis and prognosis in superficial oesophageal cancer and concluded that LVI plays an important role in the prognosis of lymph node metastasis in superficial oesophageal cancer [20]. In this study, LI could not only assess preoperative lymph node metastasis but also predict postoperative local lymph node recurrence.

With the release of a series of clinical study results [35–37], neoadjuvant therapy (neoadjuvant chemotherapy and neoadjuvant chemoradiation) is recommended by many guidelines as induction therapy for oesophageal cancer, and our clinical practice is also recommended in accordance with relevant guidelines. A meta-analysis of different neoadjuvant treatments for oesophageal cancer included 25 randomized trials involving 5272 patients, its result showed that compared with chemotherapy, neoadjuvant concurrent chemoradiation has obvious advantages in the survival of surgical treatment of resectable oesophageal cancer [38]. Our study was to explore the effect of LVI of thoracic oesophageal cancer on the prognosis of patients. Therefore, all patients receiving neoadjuvant therapy were excluded from this study, which is consistent with previous related studies [21, 39].

There are some limitations to the present study that need to be addressed. First, in our retrospective study, an inherent bias cannot be excluded. Second, we only analysed the overall positive or negative results in LI and VI, and peritumoural, invasive front and intratumoural areas should be investigated in future research. Moreover, although we use various methods to follow up patients, 7.8% of patients are still lost in the process. Nevertheless, in our study, 147 patients died of cancer but showed no clear signs of metastasis and recurrence, which might have skewed the survival analysis. Prospective studies are necessary in future work, which could potentially guide new staging methods and treatment principles of ESCC.

## Conclusion

We concluded that LVI is an important supplement to the TNM staging of ESCC and that LI and VI should be evaluated separately. LI could not only assess preoperative lymph node metastasis but also predict postoperative local lymph node recurrence.

## Data Availability

Not applicable.
